# Transitioning from cytology-based screening to HPV-based screening at longer intervals: implications for resource use

**DOI:** 10.1186/s12913-016-1375-9

**Published:** 2016-04-26

**Authors:** Megan A. Smith, Dorota Gertig, Michaela Hall, Kate Simms, Jie-Bin Lew, Michael Malloy, Marion Saville, Karen Canfell

**Affiliations:** Cancer Research Division, Cancer Council NSW, 153 Dowling St, Sydney, NSW 2011 Australia; School of Public Health, University of Sydney, Sydney, Australia; Victorian Cytology Service Ltd., Melbourne, Australia; Melbourne School of Population and Global Health, University of Melbourne, Melbourne, Australia; Centre for Epidemiology and Biostatistics, Melbourne School of Population and Global Health, University of Melbourne, Melbourne, Australia; Department of Obstetrics and Gynaecology, University of Melbourne, Melbourne, Australia

**Keywords:** Screening, Cervical screening, HPV testing, Implementation, Cervical cancer, HPV, Vaccination

## Abstract

**Background:**

Following a recent major review of cervical screening, from 2017 Australia will transition from two-yearly cytology-based screening to five-yearly primary HPV screening, with partial genotyping and direct referral for HPV 16/18 and LBC triage for other oncogenic types. Switching to a longer screening interval will result in transitional fluctuations for volumes of tests before a 'steady state' is reached for the new test volumes. This study aimed to quantify the impact of this transition on year-to-year volumes of screening and follow-up tests and procedures.

**Methods:**

Number of women screened and test volumes from 2015 to 2032 were estimated via a detailed simulation model which explicitly modelled varying screening and HPV vaccination exposure in individual birth cohorts, and fully incorporated how a relatively rapid screening program switch in 2017 would affect both women attending for routine screening and those in surveillance following an abnormality.

**Results:**

Numbers of women screened and HPV tests are predicted to fluctuate in the first screening rounds as a result of the transition to a longer screening interval (mean women screened and HPV tests 1.4 million in the first 5-year period, year-to-year fluctuation > +/−50 %; mean 1.5 million women/HPV tests in third 5-year period, fluctuation approximately +/−25 %). The extent to which this fluctuation was predicted to carry through to secondary tests/procedures was less (fluctuations of +25 %/-31 % in first 5-year period; decreasing to +8 %/-10 % by third round). HPV vaccination is predicted to counteract increases in high grade cytology results, colposcopies and precancer treatments which would otherwise occur due to population increases. Precancer treatments are predicted to drop below 2015 levels within the first few years of program switchover. Mean colposcopy volumes are predicted to be similar to 2015 levels by the third round of HPV-based screening, and also be 25–40 % lower than would have occurred in the absence of HPV vaccination.

**Conclusions:**

While numbers of women attending for screening and HPV tests are anticipated to initially fluctuate as a result of the transition to a longer recommended interval, there is expected to be less fluctuation in follow-up tests and procedures; however these will still have a significant impact on operational aspects of the screening program. Detailed modelling of the switchover process gave important insights into how volumes would be affected.

**Electronic supplementary material:**

The online version of this article (doi:10.1186/s12913-016-1375-9) contains supplementary material, which is available to authorized users.

## Background

As a result of a recent review of the National Cervical Screening Program (NCSP) in Australia (known as “Renewal”), major changes are planned [1]. Broadly, these changes involve a switch from a recommendation of two-yearly screening with conventional cytology between the ages of 18–69 years, to five-yearly screening between the ages of 25–74 years using primary testing for human papillomavirus (HPV) with partial genotyping for HPV 16/18, and liquid-based cytology (LBC) triage for women who test positive for oncogenic HPV types other than 16/18 [1]. In addition, the NCSP will change to an active call-recall system – inviting women to be screened when they turn 25 and recalling them when they are due to re-attend - rather than a reminder system, where letters are only sent to women who are 3 months overdue for their next recommended test. The organisation of the cervical screening register will also change from eight jurisdiction-specific Pap test registers (which only include women who have been screened at least once within that jurisdiction), to a single national cancer screening register, populated with whole-of-population data. These changes are expected to be implemented from May 2017, and the use of conventional cytology for cervical screening is expected to be phased out within 6–12 months via removal of the reimbursement for this test [1]. Therefore, a relatively rapid switchover from the current practice of two-yearly cytology-based screening to primary HPV-based screening is planned to occur, starting in 2017.

As part of the Renewal process, extensive evidence-based modelling of potential screening strategies was undertaken and resource utilisation estimates were made [2]. Resource utilisation estimates included the annual numbers of HPV tests, cytology tests, colposcopies, and women treated for precancerous lesions. However, these previous estimates from the model represented post-transitional ‘steady-state’ estimates. In the period after implementation, the program will be switched from a 2-year interval to a 5-year interval across all target ages within a comparatively short period, since women predominantly re-attend in a 2- to 3-year timeframe after their most recent screening test. As a result, there will potentially be fluctuations in women attending and resource utilisation due to the change in the recommended interval, and this would have implications for workforce and resource planning around the transition.

The aim of the current study was to estimate the year-by-year volumes immediately before and for the first 15 years after the introduction of the renewed program (2015–2032; approximately three screening rounds) of: number of women screened, HPV tests, LBC tests, colposcopies and precancer treatments following the implementation of the Renewed program, taking into account the different ways women transition into the new program and the impact of HPV vaccination. This was done in two ways, in order to compare the results from a simpler more intuitive versus a more detailed modelled approach. An additional comparison was made with what volumes would have been expected in the absence of a change to the screening program and/or HPV vaccination, in order to clarify which changes were due to demographic change, which were due to the screening program change and which were due to the impact of HPV vaccination, since by 2017 women aged up to 36 years will have been offered vaccination through a national publicly-funded vaccination program.

## Methods

**Fig. 1:**
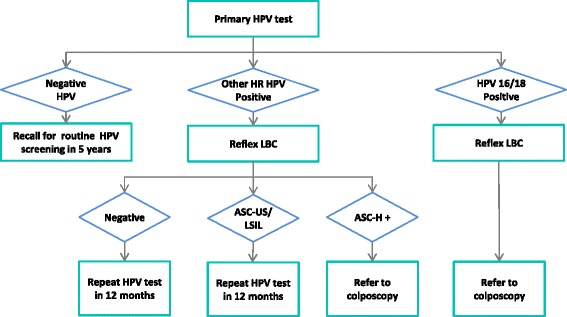
Proposed cervical screening algorithm in Australia from May 2017. Detailed clinical management guidelines are currently being developed; this algorithm is based on that recommended by the Medical Services Advisory Committee [1]. Reflex LBC is performed in all women who test positive for oncogenic HPV. For women who test positive to HPV 16/18, the LBC result does not affect the referral decision, but is used to inform colposcopy

As part of the original Renewal evaluation of primary HPV screening, we developed a detailed model of cervical screening according to the now recommended changes (“proposed pathway”; see Fig. 1), which we used in a comparison with current screening practice [2]. Management pathways for screen-positive women were based on a pre-specified protocol [3], existing guidelines [4], and expert advice from the Renewal Steering Committee [2]. Briefly, women who test negative for oncogenic HPV are recommended to return in 5 years; women who test positive for oncogenic HPV all have reflex LBC. Women who test positive for HPV 16/18 are referred for colposcopy, and the results of their reflex LBC test are used to inform colposcopy; women who test positive only for other (non-16/18) oncogenic HPV types are triaged to either immediate colposcopy or a repeat HPV test in 12 months, based on the result of their reflex LBC test. This screening model was based on a model platform developed over many years, which has been extensively validated and widely used for HPV vaccination and cervical screening policy evaluations for Australia, New Zealand, England and China [5–15]. The platform consists of multiple elements including models of sexual behaviour, HPV transmission, natural history of HPV infection, HPV vaccination, screening behaviour, management of screen-detected abnormalities including treatment of precancerous lesions, and cervical cancer treatment and survival (see Additional file 1). These elements have previously been described in detail [2].

The model incorporates detailed rescreening behaviour, informed by a previous analysis of data from the Victorian Cervical Cytology Registry (VCCR) [11], which records all cytology and histology tests undertaken in the state of Victoria (approximately a quarter of the Australian population) unless women have opted off the VCCR. This approach was able to account for some degree of early and late rescreening by women, and variation by age (see Additional file 1) [2]. Since the transitional recommendations were not yet available at the time of this analysis, for this evaluation we assumed that from 2017, several changes would occur in screening behaviour, consistent with the Renewal recommendations and the draft reimbursement schedule for cervical screening tests (which restricts testing to women aged 25–74 years and no sooner than 57 months after a negative HPV screening test, or for management of a previous abnormality) [1], since these recommendations and reimbursement restrictions are intended to alter screening behaviour. For women aged 25 or less, assumed changes include that from 2017 women aged less than 25 years would no longer initiate screening but that women aged less than 25 years who had initiated screening prior to 2017 may re-attend (for example to follow-up a recent abnormality; the simple estimates additionally considered that re-attendance by women aged less than 25 would be only half what it was prior to 2017); that all women would receive an invitation to attend for cervical screening in the year they were aged 25 years, unless they had attended for screening in the previous year; and that uptake would be fast (74 % at age 25; 83 % by age 29; consistent with current initiation patterns before age 30) [2]. We assumed that from 2017 on, all women attending for screening would be screened using HPV as the primary test with follow-up according to the proposed pathway. It was assumed that no women who were screen-negative would re-attend in the first 3 years after a screening visit (based on reimbursement restricting routine testing to within 57 months of a negative HPV test), however some early re-screening was assumed to occur in the fourth year after a negative HPV test (see Additional file 1) [2]. As there were no direct local data on screening behaviour in the context of an active call-and-recall (rather than reminder-only) system using a whole-of-population register, we used registry data from England, where a call-and-recall system was in place, to inform estimated patterns around early versus on-time versus late rescreening, using previously described methods [2, 11]. It was assumed, however, that changing the recommended interval would not alter screening behaviour in very underscreened women, so in order to take between-country differences into account, the re-attendance rates for England were modified so that the modelled proportion of women not screened for at least 7 years was consistent with that observed in Australia under current practice [2].

Table 1 summarises the scenarios examined. In the main analysis, we assumed the present reminder system would continue over the transition period; that is, that reminders to attend would only be sent to women who are 3 months overdue for their next recommended test (generally 27 months after a negative cytology result) (‘gradual change’). In this case, women already in the program would not be actively recalled for screening either before or around the time that they are due until after they have had their first primary HPV screen. In an alternative scenario, we examined the impact of an additional active ‘recall’ in 2018 as part of the transition, where all women aged 26–69 years who had previously been screened but were not screened in 2017 would be sent an invitation to attend in 2018 (ie 24 months or more after their most recent screening test). The response rate to this invitation was assumed to be relatively high; specifically, this was done by assuming that the cumulative proportion of women last screened in 2015 or 2016 who were re-screened by the end of 2018 was at least as high as it would be in response to the recall invitation sent 5 years after an HPV test (see Additional file 1). In practice, this meant that approximately 77–90 % of women aged 26–69 years who were screened in 2015 or 2016 had re-attended by the end of 2018 (range represents variation by age); and on average 90 % or more of women who were screened at least once between 2008 and 2014 had re-attended by the end of 2018. With respect to assumptions around initiating screening, in both the active recall and the gradual change scenarios, the same assumptions were made for inviting women at age 25.

**Table 1:** Summary of scenarios examined

Scenario name	Model	Vaccination?	Description
Gradual change (simple method)	Simple	No	Assumes a gradual change to the new program in 2017 (no recall invitations sent to women aged >25 until after their first HPV screening test; current system where reminders are only sent to women who are 3 months overdue for their next recommended test remains in place). Re-attendance is assumed to be equivalent to that observed under the current reminder-based system.
Active recall 2018 (simple method)	Simple	No	Assumes an active recall invitation is sent in 2018 to all women due for screening who did not attend in 2017 ie sent two years or more after their most recent cytology screening test. Re-attendance following this invitation is assumed to be at least as high as that under a call-recall invitation system after a routine HPV test.
Gradual change (detailed method)	Detailed	No	Assumes a gradual change to the new program in 2017 (no recall invitations sent to women aged >25 until after their first HPV screening test; current system where reminders are only sent to women who are 3 months overdue for their next recommended test remains in place). Re-attendance is assumed to be equivalent to that observed under the current reminder-based system.
Simple method – approximate vaccine effect	Simple	Yes	Simpler estimates assuming a gradual change to the new program in 2017 and approximating the effect of HPV vaccination: vaccine effect is applied to women born in 1981 or later (ie aged 26 or less for at least the first six months of the catch-up HPV vaccination program)
Simple method – no vaccine effect	Simple	No	Simpler estimates assuming a gradual change to the new program in 2017 but no effect of HPV vaccination (what would have been observed under the new program in the absence of HPV vaccination)
Detailed method with vaccination/Program change in 2017 with vaccination	Detailed	Yes	Detailed estimates assuming a gradual change to the new program in 2017 and taking into account the effect of HPV vaccination over time *(best and most detailed estimates for likely future resource use)*
Detailed method – no vaccine effect/Program change in 2017 – no vaccine effect	Detailed	No	Detailed estimates assuming a gradual change to the new program in 2017 but no effect of HPV vaccination (what would have been observed under the new program in the absence of HPV vaccination): this counterfactual allows examination of the effect of the screening program change and demographic change only.
Current practice with vaccination	Detailed	Yes	Detailed estimates of resource use in the absence of any change to the current program (see Additional file 1: Figure S2) but which take into account the effect of HPV vaccination: this counterfactual allows examination of the effect of HPV vaccination and demographic change only.
Current practice – no vaccine effect	Detailed	No	Detailed estimates of resource use in the absence of any change to the current program and in the absence of HPV vaccination: this counterfactual allows examination of the effect of demographic change only.

### Estimates of the number of women screened and test volumes

We used two methods to estimate the future number of women screened and test volumes. A simpler hybrid approach estimated the future number of women screened from previously observed data on attendance and patterns of re-attendance from the VCCR analysis. Model predictions of age-specific resource use per woman screened were then used to translate the expected number of women screened into expected test and procedure volumes. The detailed method involved modelling individual birth cohorts’ differing exposure to each of the current and the proposed screening program over their lives. The details of each method are described below. Both methods take into account HPV vaccination using similar assumptions around vaccine uptake, so this is described in a separate section. Both methods used Australian Bureau of Statistics (ABS) estimates for the projected population by age (Series B) to estimate the number of women invited at age 25 [16].

### Simpler (hybrid) method

The expected number of women screened was estimated starting from age-specific counts of the number of women screened in each calendar year between 2003 and 2012 (the most recent year for which published data were available) [17, 18]. From 2013 on, the number of women attending was estimated based on the same analysis of VCCR data as is incorporated into the model. This analysis described the interval-specific probability that a woman will return after a routine negative screening test (routine recall) for intervals of 1 to 10 years. A proportion of women were assumed to return at 12 months due to a positive screening test where recommended follow-up was 12 months rather than colposcopy referral based on preliminary clinical implementation data.

In order to estimate test/procedure volumes, the simpler method derived an estimated rate of tests/procedures per woman screened (“resource-use rates”) from the steady-state estimates from the detailed screening model. Age-specific resource-use rates were then applied to age-specific estimates of the number of women screened in a given year, and totals for each year obtained by summing across all ages. Age-specific resource-use rates were able to take into account differences both in the underlying risk of disease, and the probability that a woman will attend for screening; and were additionally able to take into account the impact of HPV vaccination in younger birth cohorts. Additional details are provided in Additional file 1.

### Detailed method

To obtain detailed estimates that accounted for women’s different screening histories at the time of program transition, the cohort-based screening model was configured to run separately for each birth cohort, and so that in all cases women were screened according to the current pathway prior to 2017, then according to the proposed program from 2017. HPV incidence for each birth cohort was derived from a dynamic transmission model which took into account both direct and indirect protection from HPV vaccination. Model predictions of the age-specific proportion of women screened and resource-use rates were scaled for each cohort according to the estimated resident population of Australia by age and calendar year to obtain cross-sectional outcomes [16].

### Accounting for the impact of HPV vaccination

The National HPV Vaccination Program in Australia commenced in 2007 for females, and included catch-up vaccination for females aged 12–26 years until the end of 2009 [19, 20]. Since 2010, girls aged 12–13 have been offered vaccination, with the addition of boys from 2013, including catch-up of boys aged 14–15 over 2013 and 2014. Both the simpler and detailed estimates incorporated the effect of HPV vaccination with uptake for females across different ages as observed in the current vaccination program [2]. National uptake data in males were not yet available so we assumed equivalent coverage in males offered vaccination from 2013 as achieved in females offered vaccination at the same age, based on initial state-based reports of similar uptake [21].

The original estimates from Renewal presented test volumes for two types of modelled cohort – one in the absence of a vaccination program, and the other where the women had been offered vaccination at the age of 12 years [2]. The simpler estimates accounted for the impact of HPV vaccination in the current year-by-year estimates using a hybrid approach, where there is assumed to be a vaccine effect in women born in 1981 or later (aged 26 or less throughout 2007), but no impact of vaccination in women born prior to 1981 (aged 27 years or older in 2007) (see Additional file 1). For the detailed estimates, birth cohorts were modelled individually and so directly incorporated the relevant vaccine uptake, as well as incorporating any indirect protection effects.

## Results

**Fig. 2:**
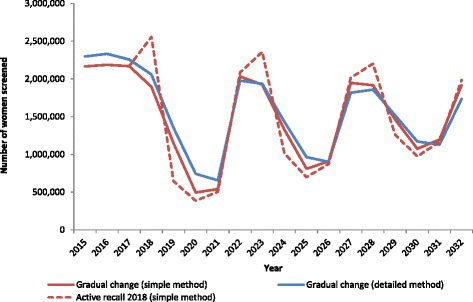
Estimated number of women attending for cervical screening, by year

Figure 2 shows the estimated number of women screened in each year from 2015 to 2032, either in the context of a more gradual change from current practice to the proposed pathway (reminders only are sent out prior to women’s first HPV test; active recall commences after a woman’s first HPV test), or alternatively a rapid transition (active recall in 2018, assuming a similar response rate to that for an active recall 5 years after a woman’s first HPV test). The estimated number of women screened was broadly similar for both the simpler and detailed methods. An active recall in 2018 is likely to substantially increase the number of women screened in 2018, and every 5 years later, compared to what would be expected with a gradual change. This was primarily due to the active call in 2018 creating a shift in attendance by women most recently screened in 2016 to predominantly re-attend 2 years later in 2018, rather attending over 2018–2019, which would have been the expected pattern based on observed screening behaviour in the context of reminders only. A sensitivity analysis was performed on the assumption that re-attendance for screening in women aged less than 25 years would halve after 2017 compared to what it would have been, in light of them no longer being in the target age range for screening. Re-attendance by women aged less than 25 years was scaled by 0.1 (ie 90 % reduction in women re-attending compared to current patterns) and 0.9 (ie 10 % reduction in women re-attending compared to current patterns); this had no appreciable impact on the number of women screened (data not shown).

**Fig. 3:**
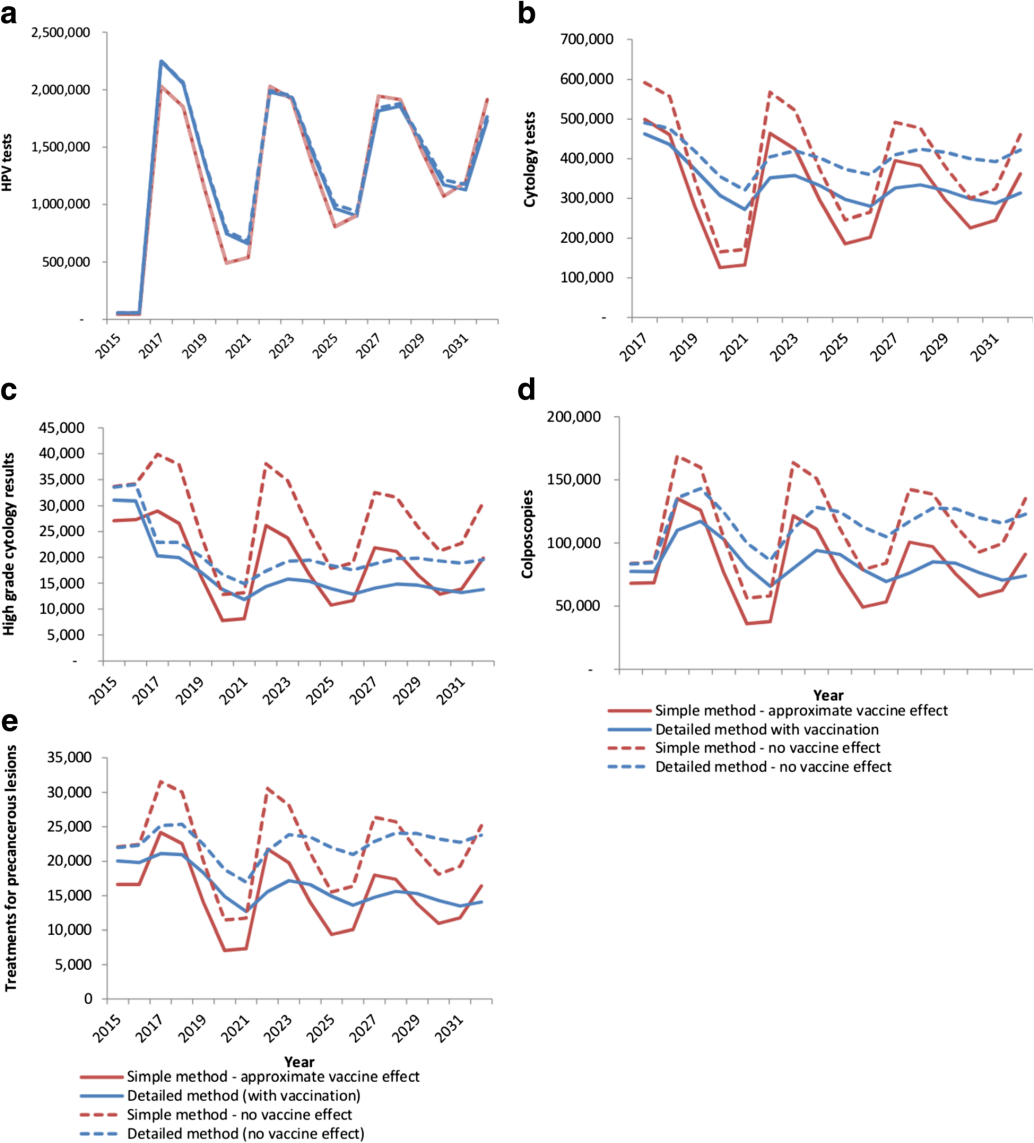
Estimated volumes of **a** HPV tests; **b** cytology tests; **c** high grade cytology results; **d** colposcopies; and **e** precancer treatments under the new proposed pathway with and without the impact of HPV vaccination: comparison of predictions from simple versus detailed method. High grade cytology results: those with a result of HSIL or ASC-H (Atypical squamous cells, possible high grade lesion) in The Bethesda System 2001; this is equivalent to possible HSIL in the Australian Modified Bethesda System 2004. High grade cytology results do not include those performed as a reflex test for women testing positive for HPV 16/18 at the primary test, nor those resulting from samples collected at colposcopy. Approximate vaccine impact = assumes rates as per cohorts offered vaccination at age 12 for all women born in 1981 or later (ie aged 26 or less throughout 2007), and rates as per unvaccinated cohorts for women born before 1981. No vaccine impact = estimates in the absence of HPV vaccination. Detailed method with vaccination = incorporates the effect of HPV vaccination (including indirect protection of unvaccinated individuals) based on observed uptake in individual birth cohorts

Figure 3 compares estimates from the simple versus detailed methods assuming in both cases that there is a gradual change to the proposed pathway (reminders only prior to first HPV test; no active recall in 2018), and indicates the variation in expected volumes which is due to the impact of HPV vaccination and for each method. Volumes are generally predicted to fluctuate over a 5-year cycle, as a consequence of the predicted fluctuations in the number of women screened. These fluctuations are predicted to decrease in magnitude with each screening round. As the primary test, the number of HPV tests was estimated to be very similar regardless of whether vaccination was taken into account or not, and the estimated volumes were similar from the simple and detailed method (Fig. 3). When vaccination is taken into account, and based on the detailed modelling, the number of HPV tests is likely to vary from ~670,000 to ~2.28 million within the first five years of the renewed program, representing a fluctuation of +59 %/-53 % compared to its mean value in the 5-year period (~1.4 million) (Table 2). By the third round (2027–2031), the fluctuation in HPV test volumes is predicted to be less, from ~1.1 million to 1.9 million, but this is still relatively large in relation to the mean value (+24 %/-25 % of the mean value of 1.5 million).

**Table 2:** Five-year averages and fluctuations in predicted volume estimates for HPV tests, cytology tests, high grade (ASC-H/HSIL) cytology results, colposcopies, and treatments for precancerous lesions, 2015–2031 (detailed method)

	Including vaccination effect				No vaccine effect			
	2015	2017–2021	2022–2026	2027–2031	2015	2017–2021	2022–2026	2027–2031
Women screened								
Mean	2,297,161	1,412,933	1,442,095	1,498,228	2,300,662	1,430,693	1,468,363	1,534,421
Min		655,625	900,795	1,128,045		683,095	942,133	1,178,039
Max		2,256,515	1,977,963	1,858,245		2,262,166	1,992,569	1,882,566
*% fluctuation (−)*		*−53.6 %*	*−37.5 %*	*−24.7 %*		*−52.3 %*	*−35.8 %*	*−23.2 %*
*(+)*		*59.7 %*	*37.2 %*	*24.0 %*		*58.1 %*	*35.7 %*	*22.7 %*
HPV tests								
Mean	54,380	1,413,004	1,442,838	1,497,588	55,857	1,430,105	1,468,317	1,532,790
Min		658,946	900,189	1,127,018		685,697	940,671	1,175,952
Max		2,248,033	1,980,721	1,857,781		2,253,235	1,994,633	1,881,146
*% fluctuation (−)*		*−53.4 %*	*−37.6 %*	*−24.7 %*		*−52.1 %*	*−35.9 %*	*−23.3 %*
*(+)*		*59.1 %*	*37.3 %*	*24.1 %*		*57.6 %*	*35.8 %*	*22.7 %*
Cytology								
Mean	2,409,923	370,206	323,456	313,008	2,418,505	412,178	391,910	408,425
Min		272,018	279,994	287,389		320,588	359,966	392,498
Max		462,288	357,152	333,685		490,507	419,585	424,015
*% fluctuation (−)*		*−26.5 %*	*−13.4 %*	*−8.2 %*		*−22.2 %*	*−8.2 %*	*−3.9 %*
*(+)*		*24.9 %*	*10.4 %*	*6.6 %*		*19.0 %*	*7.1 %*	*3.8 %*
ASC-H/HSIL cytology								
Mean	31,002	16,607	14,462	14,100	33,532	19,504	18,415	19,307
Min		11,869	12,885	13,187		14,910	17,358	18,768
Max		20,281	15,763	14,831		22,888	19,436	19,837
*% fluctuation (−)*		*−28.5 %*	*−10.9 %*	*−6.5 %*		*−23.6 %*	*−5.7 %*	*−2.8 %*
*(+)*		*22.1 %*	*9.0 %*	*5.2 %*		*17.4 %*	*5.5 %*	*2.7 %*
Colposcopies								
Mean	77,508	95,367	82,677	78,421	83,234	117,901	116,479	121,557
Min		65,633	69,454	70,464		86,276	105,079	115,722
Max		117,173	94,166	85,045		142,970	128,322	127,757
*% fluctuation (−)*		*−31.2 %*	*−16.0 %*	*−10.1 %*		*−26.8 %*	*−9.8 %*	*−4.8 %*
*(+)*		*22.9 %*	*13.9 %*	*8.4 %*		*21.3 %*	*10.2 %*	*5.1 %*
Treatments								
Mean	20,011	17,593	15,572	14,694	21,984	21,745	22,375	23,395
Min		12,680	13,604	13,507		16,945	21,002	22,763
Max		21,104	17,176	15,597		25,345	23,858	24,091
*% fluctuation (−)*		*−27.9 %*	*−12.6 %*	*−8.1 %*		*−22.1 %*	*−6.1 %*	*−2.7 %*
*(+)*		*20.0 %*	*10.3 %*	*6.1 %*		*16.6 %*	*6.6 %*	*3.0 %*

*ASC-H* atypical squamous cells, possible high grade lesion in The Bethesda System 2001; ASC-H is equivalent to possible HSIL in the Australian Modified Bethesda System 2004. High grade cytology results do not include those performed as a reflex test for women testing positive for HPV 16/18 at the primary test, nor those resulting from samples collected at colposcopy. Treatments are for cervical intraepithelial neoplasia (does not include cancer treatments)

The simple and detailed methods differed, however, in their estimates for volumes of follow-up tests and procedures. The simpler method predicted a much greater fluctuation in volumes (commensurate with the pattern for HPV tests) than the detailed method (Fig. 3). In both cases HPV vaccination was predicted to reduce the overall volumes (but not necessarily the magnitude of the fluctuations). When vaccination is taken into account, and based on the detailed modelling, the number of cytology tests, high grade cytology results, colposcopies and precancer treatments fluctuated by +8/-10 % or less by the third 5-year cycle.

**Fig. 4:**
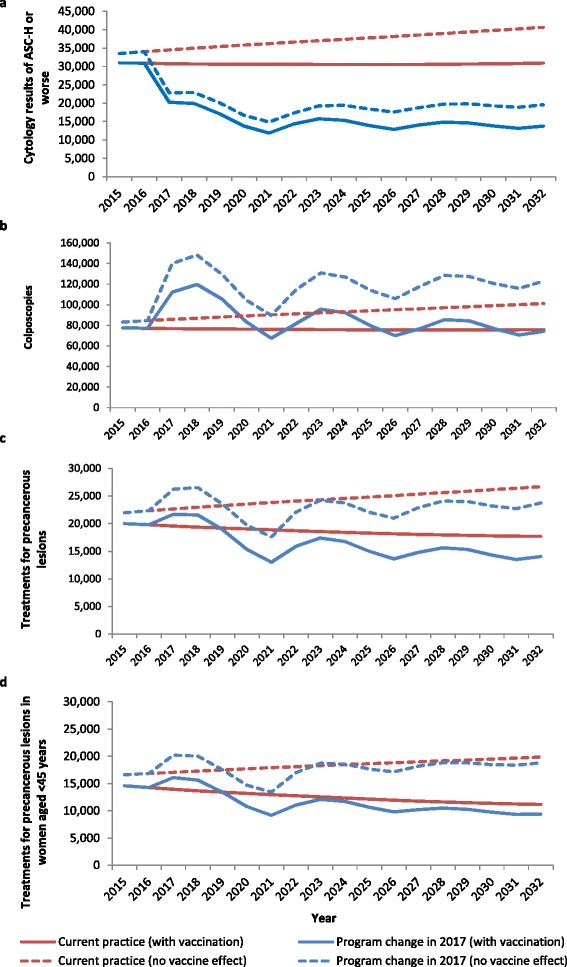
Estimated volumes of **a** high grade cytology results; **b** colposcopies; **c** precancer treatments; and **d** precancer treatments in women aged less than 45 years by year with and without the impact of HPV vaccination: comparison of current program with proposed pathway. High grade cytology results: those with a result of HSIL or ASC-H (Atypical squamous cells, possible high grade lesion) in The Bethesda System 2001; ASC-H is equivalent to possible HSIL in the Australian Modified Bethesda System 2004. High grade cytology results do not include those performed as a reflex test for women testing positive for HPV 16/18 at the primary test, nor those resulting from samples collected at colposcopy

When compared to the expected change in volumes in the absence of a change to the screening program (where changes are due to population change or HPV vaccination only), it could be seen that with or without a screening program change, HPV vaccination is predicted to counteract increases in high grade cytology results, colposcopies and precancer treatments which would otherwise be anticipated due to population increases (Fig. 4). Volumes of high grade cytology and precancer treatments are predicted to drop below 2015 levels within the first few years of program switchover, however this effect was driven by the screening program change, and would have occurred even in the absence of HPV vaccination (Fig. 4, Table 3). By the third round of HPV-based screening, mean colposcopy volumes are predicted to be similar to both current levels and to those which would have occurred in the absence of screening program change, and also be 20–35 % lower than would have occurred under either screening program in the absence of HPV vaccination. Volumes of precancer treatments were additionally considered in terms of the number which would be expected to occur in women aged less than 45 years (as precancer treatments are potentially linked to adverse obstetric outcomes [22–24] and 99.7 % of births in Australia occur in women aged less than 45 years [25]). As for precancer treatments in all ages, an initial increase in precancer treatments was predicted to occur due to the use of a more sensitive primary test; however from around 2020 onwards, the volume of precancer treatments in women aged less than 45 years is predicted to be lower than it would have been in the absence of program change (Fig. 4d).

We additionally compared the predicted volumes during the third round of screening under the renewed program (2027-2031) with those predicted by the model at steady state, consistent with the approach used for previously published findings [2], but updated compared to previous estimates to reflect the average underlying population in 2027-2031 (Additional file 1: Table S1). The mean predicted volumes during 2027-2031 were consistent with the steady state estimates in the absence of HPV vaccination, and in the context of HPV vaccination for number of women screened, and HPV tests (where the impact of vaccination on predicted volumes was relatively small). In 2027-2031, the number of cytology tests, high grade cytology results, colposcopies and precancer treatments was approaching, but had not yet reduced to, those predicted by the steady state estimates in the context of HPV vaccination. This is likely because cohorts offered vaccination did not yet make up enough of the screening population for these volumes to have reached steady state.

**Table 3:** Mean volumes of women screened, HPV tests, cytology tests, high grade or worse (ASC-H/HSIL) cytology results, colposcopies, and treatments for precancerous lesions (2015–2031) under different screening and vaccination assumptions (detailed method)

		2015	2017–2021			2022–2026			2027–2031		
	*Vaccination*	Current screening program	Current screening program	New screening program	*% increase under new program*	Current screening program	New screening program	*% increase under new program*	Current screening program	New screening program	*% increase under new program*
Women screened	*With vaccination* ^a^	2,297,161	2,429,487	1,412,933	*−41.8 %*	2,585,465	1,442,095	*−44.2 %*	2,737,383	1,498,228	*−45.3 %*
	*No vaccination*	2,300,662	2,436,693	1,430,693	*−41.3 %*	2,595,623	1,468,363	*−43.4 %*	2,750,112	1,534,421	*−44.2 %*
HPV tests	*With vaccination* ^a^	54,380	53,273	1,413,004	*2552.4 %*	50,653	1,442,838	*2748.5 %*	47,873	1,497,588	*3028.2 %*
	*No vaccination*	55,857	59,390	1,430,105	*2308.0 %*	63,263	1,468,317	*2221.0 %*	66,699	1,532,790	*2198.1 %*
Cytology	*With vaccination* ^a^	2,409,923	2,544,000	370,206	*−85.4 %*	2,702,457	323,456	*−88.0 %*	2,857,249	313,008	*−89.0 %*
	*No vaccination*	2,418,505	2,561,456	412,178	*−83.9 %*	2,727,996	391,910	*−85.6 %*	2,889,861	408,425	*−85.9 %*
ASC-H/HSIL cytology	*With vaccination* ^a^	31,002	30,661	16,607	*−45.8 %*	30,538	14,462	*−52.6 %*	30,671	14,100	*−54.0 %*
	*No vaccination*	33,532	35,399	19,504	*−44.9 %*	37,391	18,415	*−50.7 %*	39,406	19,307	*−51.0 %*
Colposcopies	*With vaccination* ^a^	77,508	76,462	95,367	*24.7 %*	75,740	82,677	*9.2 %*	75,566	78,421	*3.8 %*
	*No vaccination*	83,234	88,087	117,901	*33.8 %*	93,199	116,479	*25.0 %*	98,136	121,557	*23.9 %*
Treatments	*With vaccination* ^a^	20,011	19,214	17,593	*−8.4 %*	18,432	15,572	*−15.5 %*	17,895	14,694	*−17.9 %*
	*No vaccination*	21,984	23,254	21,745	*−6.5 %*	24,572	22,375	*−8.9 %*	25,871	23,395	*−9.6 %*

^a^HPV vaccination as has been implemented in Australia; ie females from 2007; males from 2013; catch-up vaccination for females aged 12–26 in 2007–2009 and males aged 14–15 in 2013–2014. ASC-H: Atypical squamous cells, possible high grade lesion in The Bethesda System 2001; ASC-H is equivalent to possible HSIL in the Australian Modified Bethesda System 2004. High grade cytology results do not include those performed as a reflex test for women testing positive for HPV 16/18 at the primary test, nor those resulting from samples collected at colposcopy. Treatments are for cervical intraepithelial neoplasia (does not include cancer treatments)

## Discussion

These findings indicate that as Australia transitions from a 2-year to a 5-year screening interval within a relatively short timeframe, the number of women screened each year, and consequently volumes of tests and follow-up procedures, will fluctuate. These fluctuations will likely pose resourcing and workforce challenges which need to be accounted for in planning. The requirement to scale up again after years where volumes have been lower is likely to be particularly challenging. An initial transitional active recall in 2018 would exacerbate this fluctuation effect compared to maintaining the reminder-based system until after women have had their first primary HPV test, primarily due to bringing forward re-attendance by women most recently screened in 2016 to predominantly occur 2 years later in 2018, rather spread over 2018–2019, as would have been expected in the context of reminders only. Focussing efforts on reaching unscreened or underscreened women in the years where volumes are otherwise anticipated to be lower may potentially reduce fluctuations, however this was not explicitly examined here.

While this study examined a specific situation in Australia, these findings would also be relevant to other settings planning to shift to a longer screening interval. This is likely to occur in the future, as several settings are actively considering or piloting a change from cytology-based to HPV-based cervical screening at longer intervals [26]. To our knowledge, this is the first study which has considered in detail test volumes and implications over a transition period from cytology-based to HPV-based primary screening at a longer interval.

This study was also able to separate out the effect of three major changes which will be occurring in the coming years, all of which will impact volumes of tests in the screening program – the impact of HPV vaccination, the impact of a restructure of the cervical screening program, and the ongoing impact of population growth. We found that even in the absence of any screening program change increases would be expected in case numbers for high grade cytology results, colposcopy referrals and precancer treatment as a result of population growth, but that over the next 10–15 years HPV vaccination would tend to counteract this increase. A similar pattern was also observed in the context of a screening program change from 2017, but with an overlay of fluctuating volumes due to the extension of the screening interval. However it is predicted that apart from an initial increase (in part due to a more sensitive test), colposcopy volumes would essentially drop below volumes which would have been expected in the absence of screening change or vaccination from around 2020, and that precancer treatments would drop below the levels expected in the absence of program change even after taking vaccine impact into account after around 2019.

The strengths of this study were that population-based data were used to estimate attendance for screening during the transition period, and also that it used a robust and well-calibrated model of HPV natural history, vaccination and cervical screening [2]. This model was specifically adapted during the review of the NCSP to include detailed clinical management pathways for the current and proposed new program, informed by an expert advisory committee (the Renewal Steering Committee) [2]. Other model assumptions were also reviewed by this multidisciplinary local expert advisory committee. The model was also informed by a concurrent review of the literature on screening test characteristics [27], and because it incorporated dynamic modelling of HPV transmission, was able to take into account the likely impact of HPV vaccination on volumes over this period. This was an important consideration, as the impact of HPV vaccination on estimated volumes is fairly substantial.

Our analysis also has several limitations. Firstly, for simplicity, these estimates assumed that the program would switch over at the beginning of 2017, and not from May 2017, as is currently planned. As a result, the timing of the fluctuations predicted here is potentially around six months earlier than would be observed – for example, the patterns or volumes shown here for a calendar year might in practice correspond more closely to a July-June year. The volumes are also presented at the level of a whole year, and were not able to examine fluctuations within smaller intervals. Secondly, the modelled screening behaviour used in both the simpler and detailed methods also does not take into account changes in screening behaviour since the time period of the screening register analysis, for example falling participation in younger women, particularly those who have been vaccinated [28]. The estimates were also unable to take into account any changes in screening behaviour which may occur in the lead up to the transition, for example if some women delay their routine screening visit for longer than usual in order to obtain an HPV test. It also does not take into account the potential impact of specific measures included in the new program to target unscreened and underscreened women, in particular the offer of self-collection facilitated by a health professional, because there were no directly comparable data to inform what the response to this offer will be. Thirdly, case numbers for high grade cytology results are underestimated to an extent, as they do not include those tests which are performed on women testing positive for HPV 16/18 at the primary test (as this cytology result is used to inform colposcopy, but does not alter the colposcopy referral decision), nor high grade cytology resulting from samples collected at colposcopy. Fourthly, HPV vaccine impact may have been underestimated, and if so this would potentially affect the estimated volumes of cytology (including high grade cytology), colposcopies and precancer treatments. Vaccine impact may have been underestimated by modelling uptake based on three-dose uptake reported to the national vaccination register. Notifications to the register from primary care providers was voluntary, however, and in females vaccinated as young women, survey data suggest that doses delivered through the primary care catch-up program were under-reported to the register by approximately 15 % [29]; this would potentially affect the estimated volumes of cytology (including high grade cytology), colposcopies and precancer treatments. There were some additional limitations of the simple method (which did not apply to the detailed estimates, and would contribute to the differences). Firstly, the rescreening behaviour used in the simple method to estimate the number of women screened reflect observed behaviour in women in routine screening (ie those recommended to return at the routine interval of 2 years for current practice, and 5 years for HPV-based screening), but were applied to all women. This would tend to underestimate the number of women who are re-screened within a year or two of their routine screening test, following a screen-detected abnormality. While this is partially accounted for in the estimates for HPV-based screening (ie from 2018 on) by assuming a proportion of women return at 12 months for follow-up of an initial abnormality, this is likely part of the reason for the deeper troughs in numbers of women screened compared to the detailed estimates. Secondly, the approach of using resource-use rates which are derived from the cohort model has an implicit underlying assumption that women have been screened using the proposed pathway over their screening lives (for example, that they were predominantly screened from age 25 and at 5-year intervals). This is likely the main reason for the greater fluctuation in the simple estimates compared to the detailed estimates. The results from the detailed modelling indicated that resource-use rates changed over time: the rates eventually approached the steady-state estimates extracted from the single cohort model and used for the simple estimates, but rates were different during the transition period and for the first few rounds of five-yearly screening. This was because in the initial period after transition to a new program, most women in the target population for screening will have commenced screening at a broader range of ages (from age 18, rather than predominantly at age 25) and attended more frequently than five-yearly. Finally, in the simple estimates, the impact of HPV vaccination was accounted for by using estimates for cohorts vaccinated at age 12 in 2009 for all females born in 1981 or later, who were (or will in future be) offered HPV vaccination as part of the National HPV Vaccination Program. This may overestimate vaccine impact in those vaccinated at older ages, as early analyses of linked vaccination and screening data in Australia confirm that vaccine effectiveness varies with age at vaccination [30]. Conversely, using results from a cohort offered vaccination at age 12 in 2009 will potentially underestimate the impact of vaccination in younger birth cohorts offered vaccination at age 12 in 2010 or later. The impact of vaccination is expected to be stronger in these younger birth cohorts, because indirect effects of vaccination will become stronger as more of the population is vaccinated, including males. Even considering only those cohorts vaccinated as 12 year olds (and not catch-up cohorts), those offered vaccination at age 12 in 2010 or later are likely to experience greater indirect protection from males being included in the vaccination program than the cohort modelled here, because they are more likely in the future to partner with males in cohorts who have been offered vaccination. The earliest cohort of boys offered vaccination are slightly younger than the cohort of girls offered vaccination at age 12 in 2009, whereas females are much more likely to mix sexually with similar age or slightly older males than with younger males [31, 32]. There may additionally have been some elective uptake of HPV vaccination among women who were aged 27 years or older in 2007, however this effect is likely to be small given the high cost of the vaccine and the potentially lower effectiveness at increasing age of vaccination.

We predict here that there is likely to be an initial fluctuation (increase) in colposcopies and precancer treatments after the transition to the renewed primary HPV-based screening program. This is dependent on two factors – transitional issues of changing to a longer interval, but also the improved detection (and thus higher referral) associated with the introduction of a more sensitive screening test. This increase will be mitigated by vaccine effect, which will particularly limit referrals in younger women. Therefore it should be noted that our findings for the extent of the transitional increase are sensitive to the modelled vaccine impact. However it should be noted that our modelling of vaccine impact has been based on the conservative estimate of three-dose uptake from the national register. As previously noted reporting to the register is potentially under-reported for any doses delivered through primary care, and this would particularly affect the estimates for women vaccinated as young adults in the catch-up program, who will be aged in their late 20s to 30s over the first round of screening. Some protection may also have been conferred in females who received fewer than three doses [33]. There are also a number of other assumptions about vaccine efficacy that have potential to impact our estimates. If we have somewhat underestimated the extent of vaccine-induced changes by 2017, our estimates for test volumes will be higher than those that will be eventually observed. Therefore, sentinel data now being obtained from a major trial, Compass (www.compasstrial.org.au) will be very important in quantifying expected test and procedure volumes and referral rates for the renewed program as a whole. Detailed management of screen-detected abnormalities is still to be underpinned by clinical guidelines (this process is currently underway and likely to be completed in early-mid 2016). In the absence of these guidelines, the assumptions we made were based on advice from an expert advisory committee convened as part of the Renewal review process [2]. Volumes of some tests may vary if the final guidelines differ from those assumptions. For example, volumes are likely to be affected by the post-colposcopy management of women without histologically-confirmed high grade, or with discordant results, or who are positive for non-16/18 oncogenic types, in particular those with low grade cytology. Volumes are also likely to be affected by women’s compliance with the recommendation to attend for colposcopy, which in turn could be influenced by the follow-up protocols and target timeframes under the program. The guidelines recommendation that an exit screening round be undertaken in women attending for screening when aged 70-74 years (rather than maintaining the current end age of 69, as was modelled) is also likely to affect volumes.

## Conclusions

Fluctuations in volumes of tests and procedures are predicted in the first rounds of screening as a result of the transition from a 2-year to a 5-year recommended interval in Australia. This is an effect of the switch to a longer interval within a comparatively brief timeframe, and so some degree of fluctuation could be expected in any setting where this occurs. These fluctuations in volumes are likely to pose resourcing and workforce challenges which will require specific planning. These fluctuations are expected to reduce with each successive screening round. Detailed modelling of the screening program change gave important insights into the extent of fluctuations. HPV vaccination is predicted to counteract increases in high grade cytology results, colposcopies and precancer treatments which would otherwise be anticipated over the next 15 years (regardless of screening program design) due to population increases.

## Ethics statement

This model-based study did not involve human participants; ethics approval was not required. A number of de-identified datasets were used in the prior development and calibration of the model platform which was used for this evaluation; use of these for research has been approved by the UNSW and (after dataset transfers) Cancer Council NSW Human Research Ethics Committees.

### Availability of data and materials

Not applicable - modelled analysis.

## Additional file

Additional file 1: Transitioning from cytology-based screening to HPV-based screening at longer intervals: implications for resource use. (DOCX 180 kb)
