# Supplementary material for: Transitioning from cytology-based screening to HPV-based screening at longer intervals: implications for resource use
Source: BMC Health Serv Res. 2016 Apr 26;16:147. doi: 10.1186/s12913-016-1375-9 (PMC4845438; doi:10.1186/s12913-016-1375-9)
Supplement: Additional file 1: — Transitioning from cytology-based screening to HPV-based screening at longer intervals: implications for resource use. (DOCX 180 kb) [file 12913_2016_1375_MOESM1_ESM.docx]

Supplementary Information accompanying the article:

***Transitioning from cytology-based screening to HPV-based screening at longer intervals: implications for resource use***

Contents

[Model overview 2](#_Toc442109050)

[Rescreening behaviour 5](#_Toc442109051)

[Details of the simpler (hybrid) method 6](#_Toc442109052)

[References 8](#_Toc442109053)

# Model overview

The model used in this analysis was used in the effectiveness and economic evaluation performed as part of a major review of cervical screening policy in Australia (“Renewal”) ([1](#_ENREF_1), [2](#_ENREF_2)). Model assumptions which differ from those in the policy evaluation are described in the methods section of this article. Other model assumptions are fully described in the detailed report, which is available for download from:

<http://www.cancerscreening.gov.au/internet/screening/publishing.nsf/Content/E6A211A6FFC29E2CCA257CED007FB678/$File/Renewal%20Economic%20Evaluation.pdf>

The model structure is shown in Figure S1.

Figure S1 - (a) Hybrid model of HPV transmission and vaccination; natural history of CIN and invasive cervical cancer; and cervical screening, diagnosis and treatment; (b) Schematic diagram of model structure

| (a) |  |
| --- | --- |
| (b) |  |

Figure S2 – Current National Cervical Screening Program in Australia

Data sources and management path: Based on 2005 NHMRC guidelines. 1. Includes any glandular abnormality, possible high grade endocervical glandular lesions and atypical glandular cells of uncertain significance. 3. Low grade exception women are women ≥30 years without a history of negative cytology in the past 2 years and without a histologically confirmed low grade cervical lesion in the last 2 years. 4. NHMRC guidelines recommend low grade exception women be managed with the choice of immediate colposcopy referral or repeat cytology in 6 months. Actual assigned recommendation codes from the VCCR data were used to determine the proportion assigned to each of the management paths shown in the diagram.

Figure S3 – Proposed HPV-based screening pathway


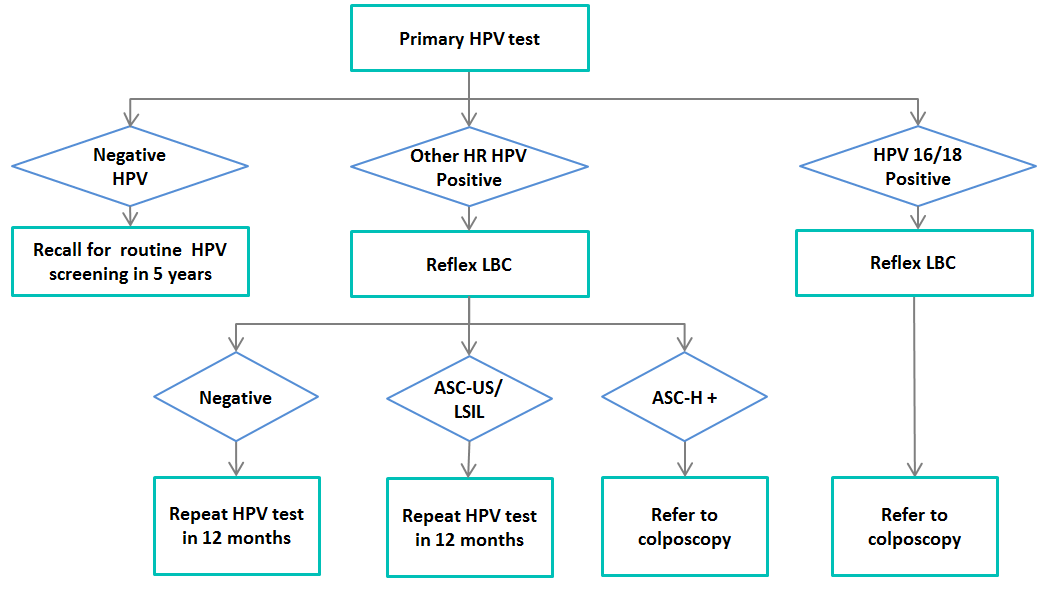


# Rescreening behaviour

The model incorporates detailed rescreening behaviour, informed by a previous analysis of data from the Victorian Cervical Cytology Registry (VCCR)([3](#_ENREF_3)). The VCCR records all cytology and histology tests undertaken in the state of Victoria (approximately a quarter of the Australian population) unless women have opted off the VCCR. This approach was able to account for some degree of early and late rescreening by women, and variation by age ([4](#_ENREF_4)). The observed cumulative re-attendance by women after a routine negative cytology test, by time since the cytology test, is shown in blue in Figure S4, based on the analysis of the VCCR data. Consistent with the recommended two-year interval and a reminder-only system (where women are only sent a reminder to attend screening if they have not attended by 27 months after their last test), most women re-attend 2-3 years after their most recent cytology test. Screening behaviour was adapted for the new recommended five-yearly interval and invitation and recall system, as shown in red in Figure S4. Early rescreening was assumed to be lower based on proposed reimbursement restrictions, which will limit reimbursement for primary HPV screening tests to those occurring no less than 57 months after a negative test, and also on the use of active invitations being sent for each screening round. Registry data from England (where an active invitation and recall system using a whole-of-population register) were used to inform patterns of early and on-time rescreening; however these patterns were always modified to account for between-country differences. Specifically, we assumed that behaviour by very underscreened women would not change and that the proportion of women not screened for more than seven years would remain the same.

Figure S4 – Cumulative probability of being re-screened by time since last negative routine screen, by age and recall period

## *Active recall in 2018*

Our primary scenarios assumed that the current system of reminders would remain in place until after a woman’s first HPV screening test, and that thereafter women would receive active invitations for subsequent rounds of HPV screening. Under the current reminder-based system, the jurisdictional screening register sends a letter to women if there is no record of a woman having repeat cytology within 27 months of their previous negative cytology test (ie when they are three months overdue). In an alternative scenario, we assumed that an active recall invitation would be sent in 2018 as part of the transitional arrangements for the program. Specifically, it was assumed that recall invitations would be sent in 2018 to all women aged 26-69 who did not attend for screening in 2017 (and thus it was at least two years since their most recent cytology screening test). For women most recently screened in 2016 or 2015 (respectively due or one year overdue for screening in 2018), we used the same assumptions about the response to this invitation as were used for the system of active call-recall invitations under the new screening program, except that on-time screening was defined as two years since last [cytology] screening test in this case, rather than five years since last [HPV] screening test. This meant that we assumed the cumulative proportion of women who re-attended within (that is, at or earlier than) two years after a cytology test was at least as high as would be expected within five years after an HPV test (ie on time or earlier in each case); and similarly that the proportion who had re-attended within three years after a cytology test was at least as high as would be expected within six years after an HPV test (ie up to one year overdue in each case); and so on for women who were more than one year overdue for screening. In practice, the most substantial impact this made was on behaviour whose most recent cytology test was two years earlier (2016). Based on observed data, under the reminder-based system, these women would have been expected to predominantly re-attend over 2018 and 2019, with approximately 33-44% and 28-36% re-attending in 2018 and 2019 respectively (range reflects variation by age). The effect of the active recall in 2018 was to bring forward screening in many of these women from 2019 to 2018. The effect of the active recall on two-year coverage over the period 2017-2018 was to increase it from an assumed 60% to 70% (ages 25-69 years, adjusted using published hysterectomy prevalence estimates ([5](#_ENREF_5))).

# Details of the simpler (hybrid) method

## *Estimates of number of women screened*

The expected number of women screened was estimated starting from age-specific counts of the number of women screened in each calendar year between 2003 and 2012 (the most recent year for which published data were available) ([5](#_ENREF_5), [6](#_ENREF_6)). From 2013 on, the number of women attending was estimated based on the same analysis of VCCR data as is incorporated into the model. This analysis described the interval-specific probability that a woman will return after a routine negative screening test (routine recall) for intervals of one to ten years. As the published data on number of women screened does not distinguish between women attending for a routine screening test and women who are being followed up at a shorter interval due to a recent abnormality, we were unable to apply different probabilities of being re-screened to women in the latter group. Instead, an age-specific proportion of women was assumed to re-attend one year after a primary HPV test, for follow-up of an initial abnormality. In the proposed pathway (still to be underpinned by clinical guidelines), women testing positive for HPV types other than HPV16/18 whose reflex cytology triage test is ASC-H or worse are likely to be referred for colposcopy, while women with LSIL or less serious results are likely to be recalled for follow-up at 12 months ([7](#_ENREF_7)), and the proportion of women who returned at 12 months was based on preliminary clinical implementation data. This partly takes into account earlier follow-up of women with a recent abnormality.

## *Estimates of test volumes*

Single-year-of-age-specific estimates were extracted from the model for HPV tests, cytology tests, high grade (ASC-H+) cytology results, colposcopies and precancer treatments, in terms of the rate of each test or procedure per woman screened (“resource-use rates”) (for example, under the proposed pathway, approximately 3.6 colposcopies are predicted to occur for every 1,000 women screened at the age of 40). These age-specific resource-use rates were derived from the model for current practice (to provide estimates for 2015 and 2016), and the proposed pathway (to provide estimates for 2017-2032). Resource-use rates are able to take into account differences both in the underlying risk of disease, and the probability that a woman will attend for screening; and were additionally able to take into account the impact of HPV vaccination in younger birth cohorts. These age-specific resource-use rates were then applied to the estimated number of women screened by single year of age, in order to estimate the number of tests which occur in each age group in a given year, and summed across all ages.

## *Accounting for the impact of HPV vaccination*

The National HPV Vaccination Program in Australia commenced in 2007 for females, and included catch-up vaccination for females aged 12-26 years until the end of 2009 ([8](#_ENREF_8), [9](#_ENREF_9)). Since 2010, girls aged 12-13 have been offered vaccination, with the addition of boys from 2013, including catch-up of boys aged 14-15 over 2013 and 2014. Both the simpler and detailed estimates incorporated the effect of HPV vaccination with uptake for females across different ages as observed in the current vaccination program ([4](#_ENREF_4)). National uptake data in males were not yet available so we assumed equivalent coverage in males offered vaccination from 2013 as achieved in females offered vaccination at the same age, based on initial state-based reports of similar uptake ([10](#_ENREF_10)).

The original estimates from Renewal presented test volumes for two types of modelled cohort – one in the absence of a vaccination program, and the other where the women had been offered vaccination at the age of 12 years. The simpler estimates accounted for the impact of HPV vaccination in the current year-by-year estimates using a hybrid approach, where there is assumed to be a vaccine effect in women born in 1981 or later (aged 26 or less throughout 2007), but no impact of vaccination in women born prior to 1981 (aged 27 years or older in 2007). For each calendar year, a set of resource-use rates is assembled by single year of age, which uses the relevant age-specific resource-use rates for either unvaccinated cohorts or cohorts offered vaccination, depending on the age of women in that calendar year. For example, the resource-use rates used in the year 2021 (when women born in 1981 attain the age of 40) employ estimates from cohorts offered vaccination for women aged up to 40 years, and use estimates from unvaccinated cohorts for women aged more than 40 years.

Table S1 – Comparison of model estimates for steady-state and over third screening round (2027-2031) which take into account detailed transition

|  | **Including vaccination effect** | **No vaccine effect** |
| --- | --- | --- |
| **Women screened** |  |  |
| Detailed transitional model (mean (2027-2031) [minimum; maximum]) | 1,498,228 [1,128,045; 1,858,245] | 1,534,421 [1,178,039; 1,882,566] |
| Steady-state estimates | 1,444,776 | 1,498,120 |
| **HPV tests** |  |  |
| Detailed transitional model (mean (2027-2031) [minimum; maximum]) | 1,497,588 [1,127,018; 1,857,781] | 1,532,790 [1,175,952; 1,881,146] |
| Steady-state estimates | 1,463,992 | 1,515,363 |
| **Cytology** |  |  |
| Detailed transitional model (mean (2027-2031) [minimum; maximum]) | 313,008 [287,389; 333,685] | 408,425 [392,498; 424,015] |
| Steady-state estimates | 266,411 | 403,457 |
| **ASC-H/HSIL cytology** |  |  |
| Detailed transitional model (mean (2027-2031) [minimum; maximum]) | 14,100 [13,187; 14,831] | 19,307 [18,768; 19,837] |
| Steady-state estimates | 12,598 | 19,086 |
| **Colposcopies** |  |  |
| Detailed transitional model (mean (2027-2031) [minimum; maximum]) | 78,421 [70,464; 85,045] | 121,557 [115,722; 127,757] |
| Steady-state estimates | 64,645 | 120,461 |
| **Treatments** |  |  |
| Detailed transitional model (mean (2027-2031) [minimum; maximum]) | 14,694 [13,507; 15,597] | 23,395 [22,763; 24,091] |
| Steady-state estimates | 12,428 | 23,110 |

# References

1. Australian Government Department of Health. National Cervical Screening Program Renewal. 2014 [updated 15/5/2014; cited 2015 21 January 2015]; Available from: <http://www.cancerscreening.gov.au/internet/screening/publishing.nsf/Content/overview-of-the-renewal>.

2. Lew JB, Simms K, Smith MA, Kang YK, Xu XM, Caruana M, et al. National Cervical Screening Program Renewal: Effectiveness modelling and economic evaluation in the Australian setting (Assessment Report). MSAC application number 1276. Canberra: Department of Health 2014.

3. Creighton P, Lew J, Clements M, Smith M, Howard K, Dyer S, et al. Cervical cancer screening in Australia: modelled evaluation of the impact of changing the recommended interval from two to three years. BMC Public Health. 2010;10:734.

4. Lew JB, Simms K, Smith M, Kang YK, Xu X, Caruana M, et al. National Cervical Screening Program Renewal: Effectiveness modelling and economic evaluation in the Australian setting (Assessment Report). MSAC Application No. 1276. Canberra2014.

5. Australian Institute of Health and Welfare. Cervical Screening in Australia 2011-2012. Cancer series no. 82. Cat. no. CAN 79. Canberra2014.

6. Australian Institute of Health and Welfare. Cervical screening in Australia 2009-2010. Cancer series 67. Cat. no. CAN 63. Canberra: AIHW2012.

7. Hammond I, Bessell T. Renewal of the National Cervical Screening Program Consultations. 2014 [updated 28/4/2014]; Available from: <http://www.cancerscreening.gov.au/internet/screening/publishing.nsf/Content/renewal-ncsp-pres>.

8. Brotherton J, Deeks S, Campbell-Lloyd S, Misrachi A, Passaris I, Peterson K, et al. Interim Estimates of Human Papillomavirus Vaccination Coverage in the School-Based Program in Australia. Commun Dis Intell Q Rep. 2008;32(4):457-61.

9. Brotherton J, Gertig D, Chappell G, Rowlands L, Saville M. Catching up with the catch-up: HPV vaccination coverage data for Australian women aged 18-26 years from the National HPV Vaccination Program Register. Commun Dis Intell Q Rep. 2011;35(2):197-201.

10. Effler P. School-based HPV Immunisation Programs: The WA Experience. Preventing Cervical Cancer conference (PCC 2015), 20-22 February 2015; Melbourne 2015.
